# 3D *in vitro* modeling of neural microenvironment through a multi-scaffold assembly approach

**DOI:** 10.1016/j.mtbio.2025.102086

**Published:** 2025-07-14

**Authors:** Cecilia Traldi, Vanessa Chiappini, Silvia Chasseur, Federica Aiello, Marina Boido, Chiara Tonda-Turo

**Affiliations:** a Department of Mechanical and Aerospace Engineering, Politecnico di Torino, Turin, Italy; b POLITO BIOMedLAB, Politecnico di Torino, Turin, Italy; c Interuniversity Center for the Promotion of the 3Rs Principles in Teaching and Research, Italy; d Department of Neuroscience "Rita Levi Montalcini", Neuroscience Institute Cavalieri Ottolenghi, University of Turin, Turin, Italy; e CNR-IPCF, National Research Council-Institute for Chemical and Physical Processes, Pisa, Italy; f Center for Instrument Sharing, University of Pisa (CISUP), Pisa, Italy

**Keywords:** 3D bioprinting, Melt electrowriting, Aligned topography, Neural stem cells

## Abstract

The engineering of *in vitro* 3D cell culture systems has emerged as promising approach to model central nervous system (CNS) intricacy with increasing physiological relevance. The fabrication of artificial microenvironments that closely resemble nervous tissue composition and architecture has provided useful substrates to promote neural cell growth and maturation under *in vivo*-like conditions; however, despite significant progress has been made in tissue mimicry, directing neural cell arrangement and connectivity in a controlled 3D environment remains extremely challenging. Here, we propose a novel approach that combines different biomaterials and biofabrication techniques to develop a multi-scaffold system mimicking distinctive features of the nervous tissue. Extrusion-based 3D bioprinting is employed to accurately position neural stem cells (NSCs) embedded in a gelatin methacryloyl hydrogel onto an aligned microfibrous polycaprolactone structure obtained by melt electrowriting. The hydrogel matrix successfully supports NSC growth within 3D bioprinted constructs, ensuring high cell viability and *in situ* NSC differentiation into neuronal and glial phenotypes. Additionally, melt electrowriting technology allows the design of a microfibrous scaffold having well-defined geometry and aligned microporosity to replicate the anisotropic characteristics of nervous tissue. The inclusion of such scaffold in the 3D bioprinted system effectively steers neural cell organization in a 3D setting, guiding neural cell elongation in a preferred direction and promoting the establishment of a functional neural network. Our approach can be used to develop more sophisticated multicellular systems, possibly reassembling specific CNS circuits within a biomimetic microarchitecture, thus offering a versatile platform for the investigation of CNS functioning and pathology.

## Graphical abstract

**Figure:**
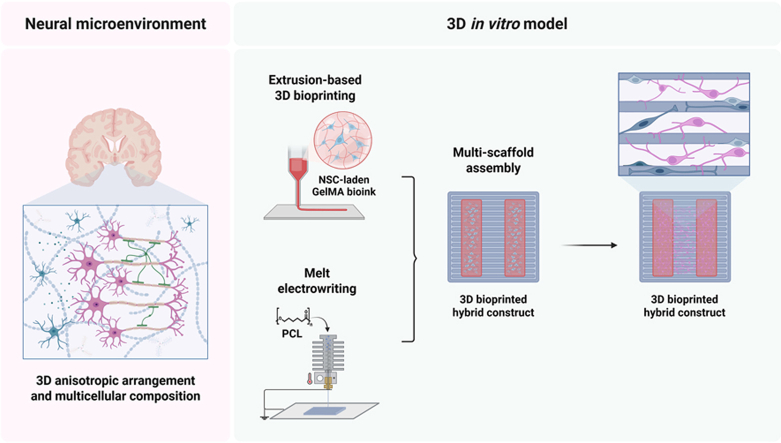

## 1 Introduction

The central nervous system (CNS) is the most functionally complex organ in the human body. Indeed, CNS is characterized by high heterogeneity at multiple scales, as it is constituted by specialized regions that coordinate and process information for enabling physiological tissue functions. At the microscale level, neural tissue has a specific cytoarchitecture, which comprises neuronal and glial cells surrounded by the extracellular matrix (ECM) and anisotropically arranged to create specific neural circuits [1,2]. During CNS development, the neural microenvironment and in particular ECM components provide guidance cues for cell migration and neurite outgrowth, resulting in the formation of aligned axon fibers, whose directionality confers structural and mechanical anisotropy to the neural tissue [3]. CNS injuries can lead to alterations of the neural microenvironment, resulting in disruption of tissue structure, permanent axon damage, and consequent impairment of sensory, motor, and/or autonomic functions [4]. Thus, modeling the distinctive features of CNS *in vitro*, in terms of cellular and extracellular composition and organization, is essential to effectively reproduce neural tissue architecture and functions toward the understanding of biological events, which occur after injuries.

Currently, natural-based soft hydrogels have been widely employed to develop 3D neural constructs, recreating artificial environments for neural cell growth and maturation, having biomimetic composition and mechanical properties [[5], [6], [7], [8]]. In particular, the application of hydrogels in 3D bioprinting allows for the automated deposition of the cell-loaded bioinks according to specific patterns, providing fine-tuning of cell arrangement at the macroscale [[9], [10], [11], [12], [13], [14], [15], [16]]. Additionally, microscale architectures have been designed to model nervous tissue morphology and alignment, by using surface patterning techniques such as lithography [[17], [18], [19]], or fiber-based substrates [[20], [21], [22], [23], [24]], which provide topographical stimuli for directing neural cell arrangement on 2D substrates. In particular, lithographic techniques have been often used to fabricate substrates having aligned microtopography that was shown to affect neural cell behavior in terms of adhesion, morphology and neurite alignment [17,18,25,26]. Accordingly, the application of aligned scaffolds in combination with microfluidic technology allowed the development of engineered neuronal circuits, by offering the possibility to directionally control axonal growth across microchannels [27]. Fiber-based topographical cues have also been widely investigated to direct neural cell spatial organization, especially by using electrospun nanofibrous mats. Indeed, fiber alignment was proven to significantly affect the arrangement and elongation of human pluripotent stem cell-derived neural cells [28], neural stem cell (NSC)-derived neurons [20,22,29] and astrocytes [30,31]. However, achieving alignment of 3D cell networks remains extremely challenging and several strategies have been proposed to promote preferential cell orientation in the modeling of both soft [[32], [33], [34], [35], [36], [37], [38], [39]] and hard [40,41] tissues, by exploring the use of synthetic polymers or natural biomolecules and different biofabrication techniques. In particular, the fabrication of composite scaffolds, obtained by layering natural hydrogel and electrospun mats, was proven to support long-term culture and directional neurite elongation of neuronal cells [37,38] as well as enhancing cell alignment in different glial cell populations [39]. Although this strategy has led to promising results in guiding neural cell behavior and arrangement, the effect of aligned topography was often limited to the fiber layer, without achieving spatial control of cells within the entire 3D structure. Indeed, electrospun mats are usually assembled in densely packed fibrous meshes with low-size porosity (from hundreds of nanometers to few micrometers [42]), which hinders cell migration within the fibrous structure and then the establishment of 3D interactions proper of the *in vivo* microenvironment. Recently, melt electrowriting (MEW) has emerged as a promising additive manufacturing technique to fabricate hierarchical microstructures and complex macro-geometry [43,44], replicating the morphology of native tissues with increasing physiological relevance [[45], [46], [47], [48]]. Compared to traditional electrospinning techniques, MEW enables the fabrication of pre-designed patterns and accurate stacking of microfibers over multiple layers, offering high control over scaffold geometry, fiber size, and construct thickness. In addition, the microscale and fully interconnected porosity of MEW scaffolds allows for cell attachment and infiltration across the microfibrous mesh (having pore size from tens to hundreds of micrometers [49,50]), thus directing cell arrangement in a 3D fashion. Despite its potential, the application of this technology in neural tissue engineering is still limited [[51], [52], [53]] and few studies reported the use of melt-electrowritten microfibers to model nervous tissue anisotropy [53]. Specifically, directional neurite extension was observed in rat phaeochromocytoma PC12 cells when cultured on polycaprolactone (PCL) microfibrous scaffolds having anisotropic geometry, confirming the guiding effect of topographical patterns provided by MEW microfibers on neuron growth [53]. On the contrary, Janzen and colleagues proposed the use of melt-electrowritten PCL scaffolds as mechanical support for primary cortical neuron 3D culture in soft matrices and demonstrated the feasibility of this approach in the study of neuron network maturity and functionality [52].

Here, we propose a novel approach relying on the integration of extrusion-based 3D bioprinting and MEW technology to create a multi-scaffold system, reassembling key features of the neural microenvironment. To the best of our knowledge, the combined use of these techniques for the development of CNS tissue models has not been reported yet. In particular, the present study aims to exploit the advantages of a photocrosslinkable gelatin methacryloyl (GelMA) bioink in supporting the growth of NSCs within an ECM-like matrix, while integrating anisotropic fiber-based topographical cues to guide neural cell maturation in an aligned 3D framework.

## 2 Materials and methods

### 2.1 GelMA synthesis

GelMA was synthesized following the protocol by Van De Bulcke et al. [54]. Briefly, Gelatin (from porcine skin, Type A, Sigma-Aldrich) was fully dissolved at a concentration of 10 % w/v in Phosphate Buffered Saline (PBS) at 50 °C. Then, methacrylic anhydride (Sigma-Aldrich) was added dropwise to the solution at 5 % v/v and the mixture was kept under vigorous stirring for 2 h at 50 °C in dark conditions. Then, the reaction was stopped by diluting three folds with PBS and the obtained solution was transferred in dialysis membranes (Spectrum™ Spectra/Por™ 2 RC Dialysis Membrane Tubing with a cut-off of 12–14 kDa) and dialyzed against deionized water at 40 °C for 7 days. Finally, the solution was lyophilized and stored at 4 °C until use. The degree of substitution (DS) of the methacrylate groups was determined by ^1^H NMR analysis.

### 2.2 NMR analysis

NMR experiments were performed on a Bruker Avance Neo spectrometer equipped with a 5 mm BBI probe operating at 700 MHz and 175 MHz for ^1^H and ^13^C nuclei, respectively. For the spectroscopic analysis, 6 mg of gelatin and 11.6 mg of GelMA were solubilized in 0.6 mL of deuterated water (D_2_O) each. In GelMA sample, 3-(trimethylsilyl)propionic-2,2,3,3-d_4_ acid sodium salt (TMSP, 1 mg/mL) was added as internal standard for the determination of the DS and for referencing the chemical shifts in all 1D and 2D experiments.

The proton NMR spectra were acquired with a 30° pulse, 128 scans, 64K data points, 2 dummy scans, and a delay of 20 s, with a spectral width of 19.84 ppm, corresponding to an acquisition time of 2.3 s. Chemical shifts were referred to TMSP (D_2_O *δ*(^1^H) = 4.79 ppm). For GelMA sample, the proton spectrum was acquired also with saturation of water signal (pulse sequence noesygppr1d).

HSQC (Heteronuclear Single Quantum Coherence) maps were acquired with 32 scans, 2048 points in F2, 16 dummy scans and a delay of 1.2 s. HSQC-TOCSY (Heteronuclear Single Quantum Coherence-TOtal Correlation SpectroscopY) maps were acquired with 32 scans, 2048 points in F2, 32 dummy scans, a delay of 1.2 s and a mixing time of 60 ms. HMBC (Heteronuclear Multiple Bond Correlation) maps were acquired with 64 scans, 1024 points in F2, 16 dummy scans and a delay of 1.2 s; Non-Uniform Sampling (NUS) was used (50 %, 128 NUS points). TOCSY (TOtal Correlation SpectroscopY) experiments were acquired with 16 scans, 2048 points in F2, 16 dummy scans, a delay of 1.5 s and a mixing time of 80 ms.

The DS of GelMA was determined by using TMSP as internal standard [55]; the signals belonging to the olefinic protons of the methacryl groups were integrated together with the methyl groups of TMSP. The following equation was used for calculating DS:

$$DS=\frac{\int methacryl}{\int TMSP}\times \frac{9H}{2H}\times \frac{n\left(TMSP\right)\left[mmol\right]}{m\left(GelMA\right)\left[g\right]}$$

### 2.3 Formulation and characterization of GelMA hydrogel

GelMA (5 % w/v) hydrogel precursor solution was obtained by dissolving lyophilized GelMA at 50 °C, followed by the addition of Lithium Phenyl(2,4,6-trimethylbenzoyl)phosphinate (LAP, TCI Europe) at a final concentration of 0.05 % w/v. The photopolymerization kinetics and the viscoelastic properties of GelMA hydrogel were investigated by rheological characterization using a modular compact rheometer (AntonPaar GmbH, MCR302) equipped with 25 mm parallel plane plates. Strain sweep tests were performed at 1 Hz frequency and strain amplitudes between 0.01 % and 500 % to primarily evaluate the linear viscoelastic region (LVE) of GelMA hydrogel. Before the test, samples were photo-crosslinked by using a visible light source at 405 nm wavelength (Prizmatix, FC-LED-405A) for 2 min. Real-time photorheology was conducted by amplitude sweep test at 1 Hz frequency and 1 % strain amplitude by irradiating samples with the aforementioned light source. All the photorheological measurements were conducted at a constant temperature of 25 °C. In addition, GelMA thermo-induced physical gelation was investigated by temperature ramp test in a range from 15 °C to 37 °C with a linear increase of 2 °C per minute, at 10 Hz frequency and 0.1 % strain amplitude. Then, the viscoelasticity of GelMA pre-gel solution within the LVE was evaluated at a constant temperature of 20 °C by performing a frequency sweep test at 0.1 % strain amplitude with frequency values ranging from 0.1 to 100 rad/s and shear rate-viscosity measurements with shear rates ranging from 0.1 to 500 s^−1^. For these tests, GelMA hydrogel precursor solution was placed on the bottom plate and kept at 20 °C for 15 min before starting measurements.

Lastly, stability tests were conducted to analyze the *in vitro* degradation kinetics of GelMA hydrogel under physiological conditions. The degradation profile was evaluated by measuring the weight loss of hydrogels incubated in PBS for different times (0, 1, 2, 7, 10, and 14 days). In particular, 180 μL GelMA hydrogel precursor solution for each sample was photo-crosslinked for 2 min and then immersed in 300 μL of PBS. At each time point, samples were washed with deionized (DI) water and freeze-dried. The percentage of weight loss (WL) was estimated for each sample (n = 3 for each time point) according to the following equation:

$$WL\;\left(\%\right)=\frac{Wo-Wt}{Wo}\times 100$$

Where *Wo* is the dry weight of the hydrogel on day 0 and Wt is the dry weight of hydrogels at the selected time point.

### 2.4 Scaffold design and fabrication by MEW

The 3D scaffold design was developed in Python to obtain a square-shaped geometry with a side of 15.40 mm and the combination of two distinct infill patterns along the z-axis. In particular, a grid pattern was selected for the bottom layer while the upper layers consisted of a parallel line-filled pattern for a total of nineteen layers. In both cases, the distance between lines was set at 0.10 mm. Then, NovaSpider v5 instrument (CIC nanoGUNE) was employed to fabricate polycaprolactone (PCL, Mw ∼ 43,000 Da; Polysciences) microfibrous scaffolds by MEW. The printing process was optimized by testing different sets of parameters having the most significant impact on the quality of the printed filaments (Table 1).

**Table 1:** Process parameters used to fabricate MEW scaffolds.

Parameter	Values
Nozzle size (mm)	0.4
Printhead temperature (°C)	95
Voltage (kV)	3.0 – 4.5
Flow rate (%)	0.4 – 5.0
Distance (mm)	7.5
Speed (mm/min)	2200

The morphology of PCL scaffolds was analyzed by using an optical microscope (Leica, M205 A). Acquired images were then processed by ImageJ software to quantify the average fiber diameter (n = 10 measurements for each sample) and distance between scaffold fibers (n = 10 measurements for each sample) in samples printed using different sets of parameters. Lastly, the mechanical behavior of MEW scaffolds was evaluated using uniaxial tensile instruments (MTS, QTestTM/10) equipped with a 50 N load cell. Measurements were performed by stressing samples along two different directions, parallel and perpendicular to fiber alignment, using a crosshead speed of 2 mm/min. Force–displacement curves were plotted to evaluate the stiffness of PCL scaffolds (n = 3 samples for each stress direction) under the two different loading conditions. In particular, stiffness values were estimated by considering the slope in the linear region of the force-displacement curves.

### 2.5 NE-4C cell line culture

NE-4C is an NSC cell line (ATCC®; LGC Standards) that can give rise to neurons and later to astrocytes. The cells were cultured in a growth medium composed of Eagle's Minimum Essential Medium (EMEM, ATCC), supplemented with 10 % fetal bovine serum (FBS, Gibco) and 1 % L-glutamine (L-Glu, Gibco). NSCs were expanded into tissue culture flasks at 37 °C in a humidified atmosphere of 5 % CO_2_.

### 2.6 3D bioprinting process

An NSC-loaded GelMA bioink (coded as neural bioink) was obtained by encapsulating NSCs at a density of 2 × 10^6^ cell/ml into sterile-filtered GelMA precursor hydrogel solution, consisting of 5 % w/v GelMA and 0.05 % w/v LAP in Dulbecco's Modified Eagle Medium (DMEM high glucose, Gibco). Before bioprinting, the bioink was loaded into a 3 ml plastic syringe and kept at 4 °C for 5 min in order to induce sol-gel transition driven by temperature. Then, an extrusion-based multi-head 3D printer (3D Discovery, RegenHU) equipped with a pressure-activated dispenser, temperature controller for the dispenser and printing platform, and integrated visible light source (405 nm wavelength, Prizmatix, FC-LED-405A) was employed to fabricate NSC-laden constructs. The printing geometries were designed through BioCAD software (RegenHU). In particular, a simple grid geometry consisting of an outer square of 10 mm side and two inner cross-shaped strands of the same length was selected to bioprint NSC-laden constructs useful for primary studies on NSC viability and differentiation (Fig. S1A). Secondly, 3D bioprinted hybrid constructs were obtained by directly bioprinting neural bioink onto MEW microfibrous scaffolds. In that case, two parallel strands with a length of 10 mm were deposited perpendicular to the PCL microfiber direction at a distance of 10 mm (Fig. S1B). Specifically, neural bioink was extruded through a 22G conical nozzle at a pressure of 30 kPa and deposited with a printing speed of 10 mm/s. The temperature of the printhead and the printing platform were set at 20 °C and 15 °C, respectively. Then, each sample was photo-crosslinked by using the integrated visible light source for 2 min. Before the bioprinting process, MEW scaffolds were sterilized by overnight immersion in 70 % ethanol solution in DI water and UV light irradiation for 1 h (30 min on each side) and then they were coated with 0.005 % Poly-L-Lysine solution.

### 2.7 NSC viability in 3D bioprinted constructs

NSC-laden 3D bioprinted constructs were cultured in NSC growth medium for up to 14 days for cell viability analyses, changing the culture medium every two days. LIVE/DEAD® (Invitrogen, ThermoFisher Scientific) assay was performed according to the manufacturer protocol to assess NSC viability in 3D bioprinted constructs after 1 day from the printing process. Specifically, the culture medium was removed and replaced by LIVE/DEAD® assay reagent solution composed of 2 μM calcein AM and 4 μM ethidium homodimer (EthD-1) solution in Dulbecco's Phosphate-Buffered Saline (D-PBS, Gibco). After incubation for 30 min at room temperature (RT), samples were washed twice with D-PBS, and cell fluorescence images were acquired using a confocal microscope (Eclipse Ti2, Nikon). The percentage of viable and dead cells was estimated using ImageJ software by considering the number of green-labeled and red-labeled cells, respectively over the total number of cells (n = 3 image fields (10X magnification) for n = 3 z-positions). CellTiter-Blue® (Promega) assay was used to quantitatively evaluate NSC viability in 3D bioprinted constructs. NSC-laden 3D hydrogels were used as control. The latter were obtained by plating NSC-loaded GelMA precursor hydrogel solution (150 μl volume) into 48-well plates, followed by visible light-induced crosslinking for 2 min. CellTiter-Blue® assay was performed at different time points (1, 3, 7, 10, and 14 days of culture) according to manufacturer protocol. Briefly, the culture medium was removed and samples (n = 3 for each condition) were incubated in CellTiter-Blue®reagent solution (1:6 dilution in culture medium) at 37 °C for 3 h. Then, fluorescence values were recorded using a multimode plate reader (SYNERGYTM HTX, BioTeK) with a 530 nm excitation/590 nm emission filter set.

### 2.8 NSC differentiation in 3D bioprinted constructs

For NSC differentiation, 3D bioprinted constructs were maintained for 3 days in NSC growth medium and then they were treated with a differentiation medium comprising DMEM, 10 % FBS, and 2 % L-Glu, supplemented by 20 × 10^−6^ M retinoic acid (RA, Sigma-Aldrich) for 4 days. The differentiation medium was then replaced with a maintenance medium composed of DMEM, 10 % FBS, and 2 % L-Glu. Culture media were changed every two days.

### 2.9 Immunofluorescence analysis

Immunofluorescence staining was carried out to investigate NSC differentiation in 3D bioprinted constructs over time (0, 14, 21, 28 days from RA induction) and assess the presence of phenotype-specific markers in hybrid 3D bioprinted constructs after 21 days from RA induction. Firstly, bioprinted samples were fixed with 4 % paraformaldehyde solution in PBS (PFA, Alfa Aesar) for 40 min at RT, and then permeabilized with 0.2 % Triton X-100 (Sigma-Aldrich) solution in PBS for 10 min. Afterwards, samples were blocked with 2 % Bovine Serum Albumin (BSA, Sigma-Aldrich) solution in PBS for 1 h at RT and incubated with primary antibodies (1:500 mouse anti-Microtubule Associated Protein-2 (MAP2) MAB3418, Sigma-Aldrich; 1:500 rabbit anti-Glial Fibrillary Acidic Protein (GFAP), Z0334, Agilent; 1:100 rabbit anti-β-Tubulin III (βIIITub), T2200, Sigma-Aldrich; 1:200 mouse anti-Nestin, MAB353, Sigma-Aldrich; diluted in 1 % BSA solution in 0.1 % Tween 20 (Sigma-Aldrich) solution in PBS (T-PBS)) overnight at 4 °C. After rinsing with 1 % BSA in T-PBS, samples were incubated with secondary antibodies (1:1000 Alexa Fluor 488 goat anti-mouse, Invitrogen; 1:500 Alexa Fluor 555 goat anti-rabbit, Invitrogen; 1:1000 Cy5 goat anti-mouse, Invitrogen; diluted in 1 % BSA solution in T-PBS) for 1.5 h at RT in dark conditions. Samples were washed with 1 % BSA in T-PBS and cell cytoskeletons were then stained by incubating in fitc-Phalloidin (Flash Phalloidin Green 488, BioLegend; diluted 1:60 in 1 % BSA in T-PBS) solution for 40 min at RT to evaluate the effect of PCL fibers on neural cell arrangement in 3D bioprinted hybrid constructs. Lastly, samples were rinsed with PBS and cell nuclei were stained by incubating in 4′,6-Diamidino-2-Phenylindole, Dihydrochloride (DAPI, Invitrogen; diluted 1:1000 in PBS) solution for 10 min at RT in the dark. Before the imaging, 3D bioprinted samples were mounted on glass coverslips by using Fluoromount™ Aqueous Mounting Medium (Sigma-Aldrich). Fluorescence imaging was performed using confocal microscope (Eclipse Ti2 confocal microscope, Nikon). All images were processed and analyzed with ImageJ software. Specifically, the assessment of cytoskeleton orientation on PCL microfibers was evaluated by using ImageJ Directionality plugin.

### 2.10 Western blot analysis

NSC differentiation in 3D bioprinted constructs was evaluated by Western blot analysis. Total protein was extracted from 3D bioprinted constructs and hybrid 3D bioprinted constructs at different time points (0, 14, 21, 28 days from RA induction and 21 days from RA induction, respectively). Specifically, 3D bioprinted constructs (n = 2 for each protein sample) were incubated in 0.05 % w/v collagenase (Collagenase from Clostridium histolyticum, Sigma-Aldrich) solution in DMEM for 1 h to degrade GelMA hydrogel. Then, cells were collected by centrifugation at 1000 rpm for 7 min at 4 °C and were suspended in ice-cold D-PBS. That procedure was repeated three times to completely remove hydrogel content. In the case of hybrid 3D bioprinted constructs, samples (n = 2 for each cell lysate) were firstly incubated in trypsin (Trypsin-EDTA 0.25 %, Gibco) solution to collect cells from PCL fibers and then treated with collagenase solution as previously described. Cell lysates (n = 3 for each condition) were obtained by incubating cells 1X RIPA solution in DI water (RIPA Lysis Buffer 10X, Sigma-Aldrich) supplemented by 1 mM PMSF, 1 mM DTT, 2 mM sodium orthovanadate (ThermoFisher Scientific) and 1X complete™ Protease Inhibitor Cocktail (Sigma-Aldrich), followed by sonication, and centrifugation at 14000 rpm for 20 min at 4 °C. Supernatants were collected and protein concentration was determined by Pierce™ BCA Protein Assay Kits (ThermoFisher Scientific). Protein denaturation was performed by adding NuPAGE® LDS Sample Buffer supplemented with NuPAGE® Sample Reducing Agent (ThermoFisher Scientific) to protein samples and heating at 95 °C for 5 min. Subsequently, proteins were separated by sodium dodecyl sulfate polyacrylamide gel electrophoresis (SDS-PAGE) using 4–15 % Mini-PROTEAN® TGX™ Precast Protein Gels (Bio-Rad) and transferred to Trans-Blot® Turbo™ mini nitrocellulose membranes (Bio-Rad) using a Trans-Blot® Turbo™ transfer System (Bio-Rad). After rinsing membranes with T-PBS, EveryBlot Blocking Buffer (Bio-Rad) was used to prevent unspecific antibody binding and incubated for 30 min at RT. The membranes were then incubated overnight at 4 °C with primary antibodies (1:1000 mouse anti-MAP2, MAB3418, Sigma-Aldrich; 1:1000 rabbit anti-GFAP, Z0334, Agilent; 1:1000 mouse anti-Nestin, MAB353, Sigma-Aldrich; 1:500 rabbit anti-βIIITub, T2200, Sigma-Aldrich; 1:1000 mouse anti-Vinculin, V9141, Sigma-Aldrich; diluted in 2 % non-fat dry milk solution in T-PBS). After washing three times with T-PBS, membranes were incubated for 1 h with secondary antibodies (HRP Conjugate Goat anti-Mouse, Biorad; HRP Conjugate Goat anti-Mouse, Biorad; diluted 1:10000 in T-PBS). Lastly, membranes were rinsed three times with T-PBS, and protein bands were visualized with Clarity™ or Clarity™ max Western ECL Blotting Substrates Western ECL (Bio-Rad) using the ChemiDoc™ imaging system (Bio-Rad). Image analysis was conducted using ImageJ software to measure the intensity of protein bands (Fig. S2). The levels of each protein were normalized to the intensity values of vinculin (loading control).

### 2.11 Scanning electron microscopy

Scanning electron microscopy (SEM) was performed to analyze the morphology of MEW scaffolds and evaluate neural cell interaction with PCL microfibers in hybrid 3D bioprinted constructs. Before the analysis, cellularized samples were dehydrated by incubation in ethanol solutions at increasing concentrations (from 30 % to 100 % ethanol in DI water). Then, all samples were coated with a thin platinum layer and images were acquired at different magnifications using a scanning electron microscope (Tescan Vega).

### 2.12 Calcium imaging

Calcium imaging was performed on hybrid 3D bioprinted constructs at 21 days from RA induction. Samples were incubated for 1 h at 37 °C with labeled calcium indicators (Fluo-4, AM, cell permeant, Invitrogen) in Hanks' Balanced Salt solution (HBSS, MP Biomedicals). Before the analysis, samples were rinsed with HBSS and transferred on glass coverslips. Time-lapse recordings were acquired for a period of 5 min at 37 °C using confocal microscope (Eclipse Ti2 confocal microscope, Nikon) and post-processed with NIS-Elements software (Nikon) and ImageJ software. Specifically, changes in fluorescence intensity (ΔF/F_0_) as a function of time were analyzed in representative regions of interest (ROIs) to detect spontaneous calcium flux in neural cell populations.

### 2.13 Statistical analysis

Graphpad Prism 10 was used for statistical analyses and to graph data which are presented as mean ± standard deviation (SD). Statistical significance in two-group comparisons was measured by unpaired *t*-test while in multiple-group comparisons it was evaluated by one-way ANOVA. A P value of 0.05 or less was selected as the level of significance with (∗) p < 0.05, (∗∗) p < 0.01, (∗∗∗) p < 0.001, (∗∗∗∗) p < 0.0001.

## 3 Results

### 3.1 GelMA synthesis and NMR analysis

GelMA was synthesized via a covalent reaction between methacrylic anhydride and the amine and hydroxyl groups of gelatin. The correct functionalization and the DS were assessed by proton (^1^H) NMR analysis.

**Fig. 1:**
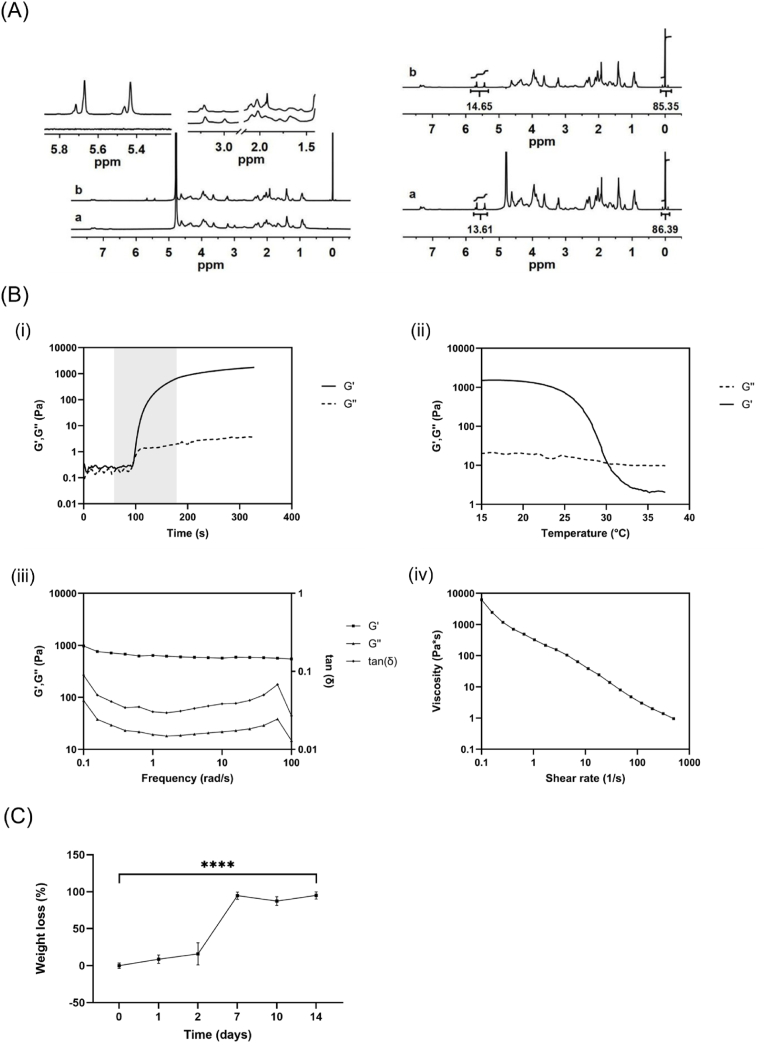
Synthesis and characterization of GelMA hydrogel. **(A)** (left) ^1^H NMR (700 MHz, D2O, 298 K) spectra of unmodified gelatin (a) and GelMA (b); (right) ^1^H NMR (700 MHz, D2O, 298 K) spectra of GelMA without (a) and with presaturation (b) for quantitative analysis. **(B)** Real-time photorheology by amplitude sweep test (i), rheological analysis by temperature ramp test (ii), frequency sweep test (iii), and viscosity as function of shear rate (iv). **(C)** Analysis of GelMA degradation kinetics under physiological conditions. Data are presented as mean ± SD (n = 3). One-way ANOVA with Tukey's multiple comparisons test: ∗∗∗∗p < 0.0001.

In the ^1^H NMR spectrum of GelMA, a new set of signals with respect to raw gelatin was found in the spectral region between 5.75 ppm and 5.40 ppm (Fig. 1A), together with a singlet signal centered at 1.92 ppm. These resonances were attributed to the double bonds and the methyl protons of methacrylamide groups bound to the amino residues of gelatin, respectively. The derivatization process also determined variations in the spectral profile of gelatin, in particular, the appearance of new signals at 3.25 ppm and 1.58 ppm, and the reduction in intensity of the signal centered at 3.01 ppm (Fig. 1A), associated to modification of the amino acids involved in the methacryloylation process.

The long-range ^1^H-^13^C correlations observed in the HMBC map showed that the protons of methacrylamide groups correlate with an ester carbon resonating at 175.0 ppm, as already observed for methacrylic anhydride (Fig. S3). An additional long-range correlation was observed between this carbon and the proton resonance centered at 3.25 ppm, belonging to the derivatized gelatin chain as presumed from the analysis of proton spectra. This long-range correlation confirmed the attachment of the methacrylamide group to the gelatin molecular structure.

The derivatization process was investigated in depth by analyzing the ^1^H-^13^C correlations through one chemical bond in the HSQC maps of GelMA, in comparison with underivatized gelatin (Fig. S4). This analysis allowed identifying the main amino acids present in raw gelatin and those that, due to methacryloylation, underwent chemical shift variation [55].

In Table S1, the list of the main amino acids identified in underivatized gelatin is reported. In addition to HSQC, HSQC-TOCSY experiment was carried out (Fig. S5); this NMR experiment provided a better resolution of the cross-peaks detectable in TOCSY experiments by exploiting the larger dispersion of carbon chemical shifts [56,57], thus helping in the identification of the amino acids.

Besides the two signals already observed in the proton spectrum of GelMA at 3.25 ppm (CH_2_, ^13^C at 42.7 ppm) and 1.58 ppm (CH_2_, ^13^C at 31.4 ppm), a signal at 3.78 ppm (CH, ^13^C at 73.0 ppm) and the proton pair at 3.37/3.28 ppm (CH_2_, ^13^C at 48.4 ppm) were identified in the HSQC map. The protons at 3.25 ppm and 1.56 ppm correlate, in the TOCSY map (Fig. S6), to resonances centered at 4.33 ppm, 1.86 ppm, 1.77 ppm and 1.40 ppm, which were assigned to modified lysine by comparison with the literature [55]. The methylene group resonating at 3.37 ppm and 3.28 ppm gave a correlation with the methine group centered at 3.78 ppm; this set of signals was attributed to the ε-CH_2_ and the δ-CH of modified hydroxylysine (Table S2). Finally, the DS of GelMA was calculated from the ^1^H NMR spectrum recorded in the presence of TMSP as internal standard, by using the equation reported in section 2.2. There are different approaches for the determination of the DS of GelMA via NMR described in the literature. The content of phenylalanine in unmodified gelatin can be used as internal standard for quantitative analysis [58,59] or the calculation of the DS can rely on the determination of the decrease of lysine in GelMA with respect to unmodified gelatin [60]. However, both methods require the quantification of amino acids (phenylalanine and lysine) present in raw gelatin. The approach used in this study, on the contrary, did not require this information since the standard was added to the sample.

The ^1^H NMR spectra of GelMA with and without presaturation of the solvent signals were analyzed, in order to estimate the impact of the (mild) presaturation on the quantitative analysis. As reported in Fig. 1A, the integrated areas calculated in the two spectra were comparable; DS was calculated as equal to 0.21 mmol/g and 0.23 mmol/g in the non-presaturated and in the presaturated spectrum, respectively.

### 3.2 Characterization of GelMA hydrogel rheological behavior and stability

GelMA hydrogel (5 % w/v GelMA and 0.05 % w/v LAP in PBS) was characterized in terms of rheological behavior to investigate its photopolymerization kinetics and viscoelastic properties within the LVE (Fig. S7). In particular, photorheological measurements revealed that GelMA hydrogel displayed a fast sol-gel transition after 2 min exposure to visible light irradiation, demonstrated by the increment in storage modulus (G’) values until reaching a stable plateau at around 1 kPa (Fig. 1B). The results of the temperature ramp test confirmed the thermoresponsive features of GelMA solution, which showed a typical gel-like behavior (G’ > G’’) before the crossover point (G’ = G’’), and liquid phase (G’’ > G’) at higher temperatures (Fig. 1B). In particular, a plateau in G’ and G’’ values was detected below 20 °C, suggesting hydrogel stability in that range of temperatures. The viscoelasticity of GelMA pre-gel solution after temperature-induced physical gelation was investigated by frequency sweep test at 20 °C, which highlighted the prevalence of elastic response over the viscous one (tan *δ* (G″/G′) < 1) within the range of tested frequencies. In addition, shear-viscosity measurements at 20 °C showed a decrease in viscosity values in response to increasing shear rates (Fig. 1B), thus confirming the shear-thinning behavior of GelMA pre-gel solution after physical gelation.

Lastly, the analysis of the degradation profile (Fig. 1C) of GelMA hydrogel revealed the presence of initial hydrogel stability after photo-crosslinking with no statistically significant differences within WL values in the first two days. However, a significant increment in WL values was observed after 7 days, resulting in complete hydrogel degradation within the observed period.

### 3.3 Fabrication of aligned microfibrous scaffold

**Fig. 2:**
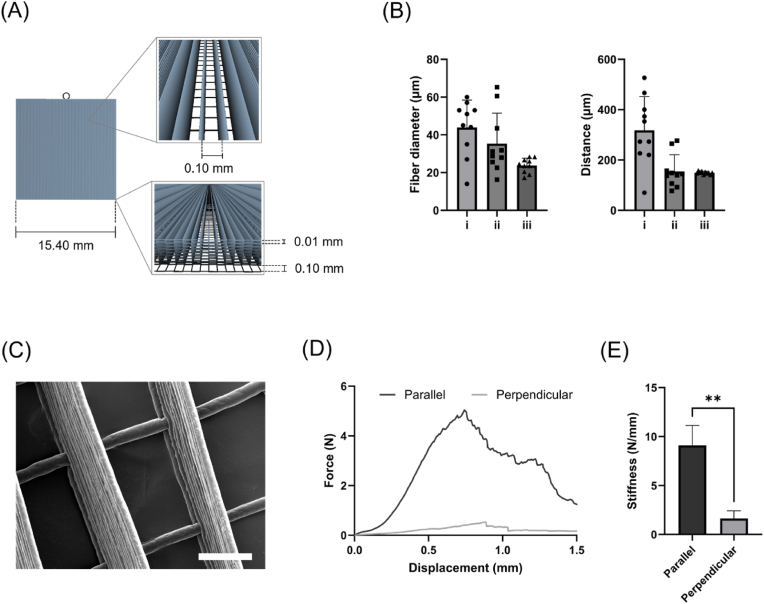
Fabrication of aligned microfibrous scaffold by MEW. **(A)** Schematics of scaffold design. **(B)** Dimensional analysis of samples fabricated with different sets of parameters: i (4500 V voltage, 3 % flow rate), ii (3030 V voltage, 1 % flow rate), iii (3030 V voltage, 0.8 % flow rate); data are reported as mean ± SD (n = 10). **(C)** Representative SEM image of MEW scaffold fabricated with the optimal set of parameters (scale bar = 50 μm). **(D)** Force-displacement curves obtained by uniaxial tensile test for samples stressed along a direction parallel and perpendicular to fiber alignment. **(E)** Comparison between stiffness in samples stressed along a direction parallel and perpendicular to fiber alignment confirming anisotropy. Data are presented as mean ± SD (n = 3). Unpaired *t*-test: ∗∗p < 0.01.

MEW was used to fabricate a PCL microfibrous scaffold with aligned geometry. The schema of the scaffold design is illustrated in Fig. 2A, highlighting the presence of a single bottom layer with a grid pattern and the following layers constituted by fibers stacked in the same direction. The morphology of the printed scaffolds was analyzed by optical microscopy to evaluate the effect of printing parameters on scaffold shape fidelity. Fig. S8 shows representative images for PCL scaffolds obtained by varying the set parameters in the ranges reported in Table 1. Differences in terms of printing accuracy and filament quality were observed and quantified by dimensional analysis. In general, samples produced with lower voltage and flow rates presented higher filament stability and fewer edge defects. Indeed, an increase in scaffold fiber diameter and distance was observed in the case of higher voltage, associated with a high variability of the measured values (Fig. 2B). In addition, decreasing the flow rate at constant voltage resulted in a reduction of fiber diameters and the bridging phenomenon, identifiable in the collapsing of adjacent fibers (Fig. S8). Scaffolds printed with the optimal set of parameters displayed a regular mesh of aligned fibers, having a diameter of 24 ± 4 μm and spaced of 150 ± 6 μm. The morphology of the scaffolds with higher shape fidelity was also observed by SEM (Fig. 2C, Fig. S9), which confirmed the regularity and uniformity of scaffold geometry, in terms of printed filaments and macroporosity.

Moreover, the mechanical behavior of MEW scaffolds was characterized through uniaxial tensile test. Fig. 2D shows the force-displacement curves obtained by stressing samples along a direction parallel and perpendicular to fiber alignment. In both cases, PCL scaffolds exhibited near-linear elastic behavior, until undergoing progressive ruptures due to fiber disassembly. However, a statistically significant difference in scaffold stiffness was observed between the two stress conditions (Fig. 2E), as samples loaded in a direction parallel to the fiber alignment displayed a stiffness value more than five-fold higher compared to the ones loaded in the perpendicular direction. These results demonstrated the anisotropic characteristics of MEW scaffolds, both in terms of structural properties and mechanical response.

### 3.4 3D bioprinting of NSC-laden constructs

**Fig. 3:**
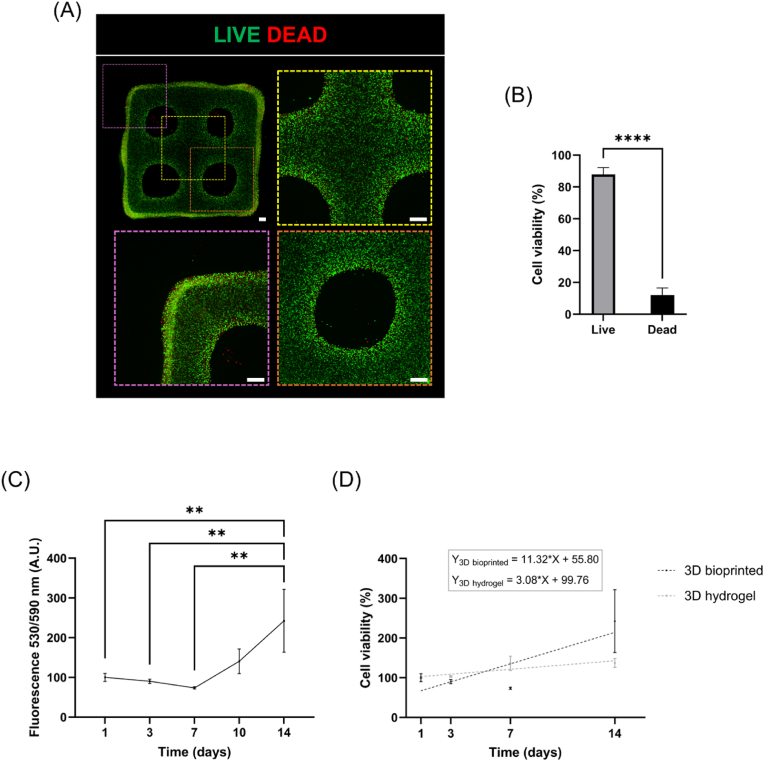
Investigation of NSC viability in 3D bioprinted constructs. **(A)** Representative images of LIVE/DEAD® assay in 3D bioprinted constructs (scale bar = 500 μm) and **(B)** semi-quantitative analysis of cell viability after 1 day from the printing process; data are reported as mean ± SD (n = 3 image fields (10X magnification) for n = 3 z-positions). Unpaired *t*-test: ∗∗∗∗p < 0.0001. **C)** Quantitative evaluation of NSC viability by CellTiter-Blue® assay up to 14 days of culture (fluorescence values are normalized to day 1) and **(D)** comparison of the viability profile of NSCs encapsulated in 3D bioprinted construct (3D bioprinted) and 3D GelMA hydrogels (3D hydrogel). Simple regression analysis on fluorescence values normalized to day 1. Data are presented as mean ± SD (n = 3 biological replicates). One-way ANOVA with Tukey's multiple comparisons test: ∗∗p < 0.01. (For interpretation of the references to color in this figure legend, the reader is referred to the Web version of this article.)

NSC-laden constructs were obtained by bioprinting neural bioink in a grid pattern to perform primary studies on the growth and differentiation of encapsulated cells. Firstly, a semi-quantitative analysis of cell viability was conducted by LIVE/DEAD® assay. As shown in Fig. 3, a high percentage of green-labeled cells was observed after 1 day of culture, while a few number of red-labeled cells were detected (87.86 ± 4.37 % of viable cells vs 12.14 ± 4.37 % of dead cells) (Fig. 3B, Fig. S10), suggesting that the printing process did not particularly affect NSC viability. In particular, live cells appeared uniformly distributed within the 3D bioprinted constructs, while dead cells were mainly localized in proximity to the filament edges (Fig. 3A). In addition, CellTiter-Blue® assay was used to measure cell viability with time and quantitatively assess the growth profile of embedded NSCs. Fig. 3C reports the detected fluorescence values for cells cultured in 3D bioprinted constructs up to 14 days, where higher fluorescence intensity indicates a higher percentage of viable cells. In particular, no statistically significant decreases were noticed after 7 days compared to day 1, while a significant increase in cell viability was observed from day 7 to day 14. In addition, the viability profile of 3D bioprinted cells was compared to the one of cells encapsulated in 3D hydrogels over 14 days of culture (Fig. 3D). According to the data, NSCs exhibited a greater viability in 3D bioprinted constructs compared to the ones encapsulated in 3D hydrogels as confirmed by the increment in slope values of the corresponding regression lines.

**Fig. 4:**
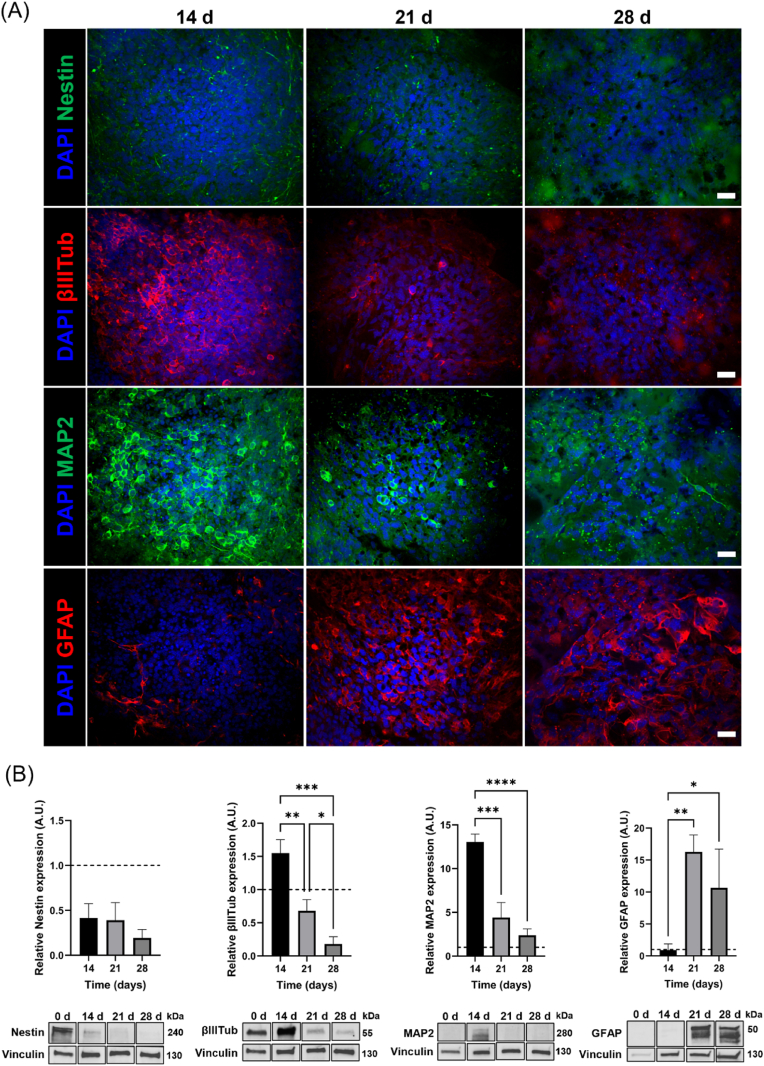
Investigation of NSC differentiation in 3D bioprinted constructs. **(A)** Representative immunofluorescence images for stemness-related (nestin), neuronal (βIIITub and MAP2), and astroglial (GFAP) markers in NSC-laden 3D bioprinted constructs at 14, 21, and 28 days of differentiation culture (scale bar = 20 μm). **(B)** Immunoblotting analysis of nestin, βIIITub, MAP2 and GFAP: protein level quantifications and representative densitometries are shown for days 14, 21, and 28 of cell differentiation. Protein levels are normalized to Vinculin (used as the loading control) and expressed relative to day 0 (dotted line). Data are presented as mean ± SD (n = 3 biological replicates). One-way ANOVA with Tukey's multiple comparisons test: ∗p < 0.05; ∗∗p < 0.01; ∗∗∗p < 0.001; ∗∗∗∗p < 0.0001.

Secondly, *in situ* differentiation of NSCs in 3D bioprinted constructs was investigated by analyzing the expression of phenotype-specific markers up to 28 days from RA induction (Fig. 4A, Fig. S11). Immunofluorescence analysis demonstrated that encapsulated cells expressed both neuron and astrocyte-specific markers after 21 days of differentiation culture (Fig. 4A). Specifically, immunofluorescence staining for neuronal-specific cytoskeleton proteins βIIITub and MAP2 at 14 days revealed the presence of large neuronal clusters, comprising early-differentiated and mature neurons, respectively. A high number of GFAP-expressing astrocytes was observed in proximity to neuronal clusters from day 21, confirming NSC differentiation into astroglial cells. In addition, a percentage of undifferentiated Nestin^+^ cells was detected at all the time points, significantly decreasing over time. Accordingly, the same trend was confirmed by Western blot analysis. Fig. 4B reports the relative intensities of protein bands for stemness-related (nestin), neuronal (βIIITub, MAP2), and glial (GFAP) markers within 28 days of differentiation culture. As expected, nestin expression was down-regulated at all the time points compared to day 0 (dotted line), thus confirming a numerical decrease in the immature neuronal population. Neuronal differentiation was demonstrated by the high levels of βIIITub and MAP2, especially on day 14. On the contrary, GFAP resulted in being highly expressed at 21 and 28 days. Interestingly, observing the time course of protein expressions, a decrease in neuronal-specific proteins was observed with the progressive increase of GFAP levels, suggesting NSC over differentiation into glial cells at late stages, as expected for this cell line. For this reason, subsequent biological characterizations have been performed considering 21 days of differentiation culture as the main time point for cell analyses.

### 3.5 Development of a 3D neural network by multi-scaffold assembly approach

**Fig. 5:**
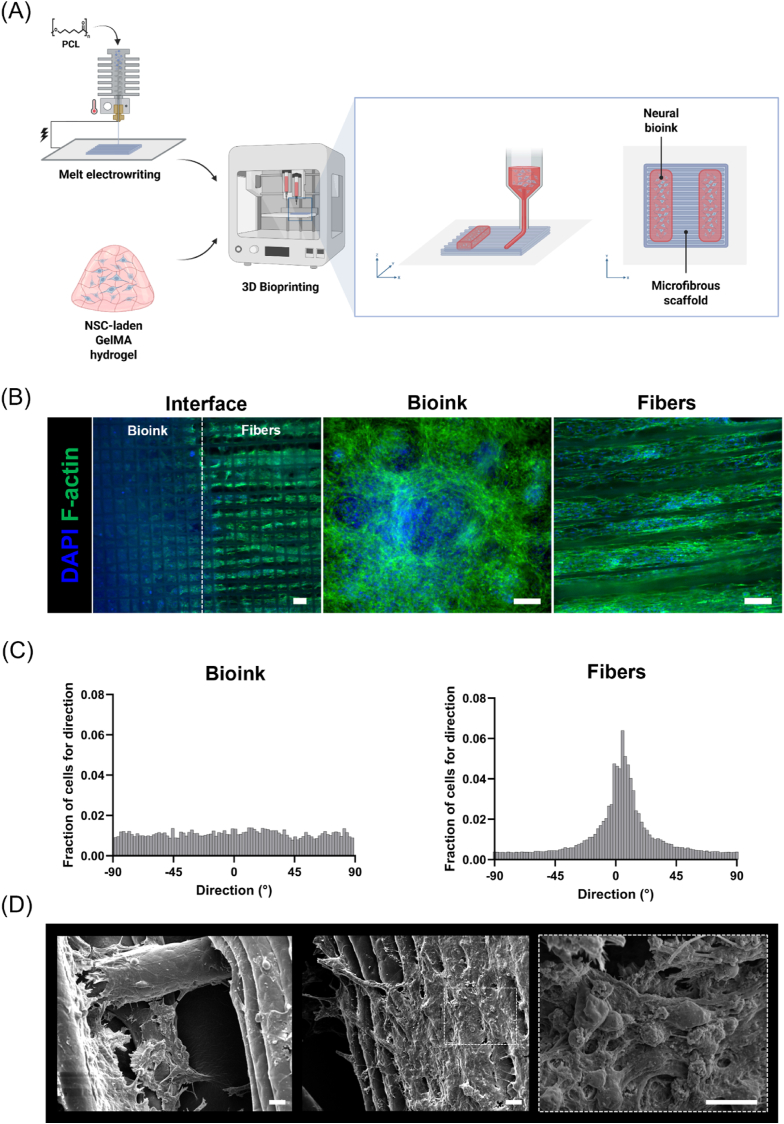
Evaluation of NSC behavior in 3D bioprinted hybrid constructs. **(A)** Schematic description of the multi-scaffold system, comprising two bioprinted lateral neural bioink compartments and a central microfibrous compartment obtained by MEW (Created with BioRender.com). **(B)** Representative fluorescence images of cytoskeleton staining in cells located at the interface between bioink and microfibers, within the bioink and along microfibers after 21 days of differentiation culture (scale bar = 100 μm). **(C)** Distribution of neural cell orientation within the bioink and microfiber compartments. **(D)** Representative SEM images of neural cells on PCL microfibers after 21 days of differentiation culture (scale bar = 10 μm).

3D bioprinted hybrid constructs were fabricated by directly extruding neural bioink on the MEW structure, thus creating a multi-scaffold system, comprising two lateral neural bioink compartments and a central aligned microfibrous compartment (Fig. 5A). The obtained constructs were maintained in differentiation culture and neural cell response was evaluated after 21 days from RA induction. Firstly, neural cell arrangement within hybrid constructs was investigated by fluorescence imaging for cell cytoskeletons in the different model compartments (Fig. 5B). Extensive migration of encapsulated cells was observed at the edge of the bioink towards PCL fibers, reaching complete colonization of the constructs after 21 days. In particular, cell infiltration uniformity within the 3D constructs was verified by fluorescence z-stack acquisition, which underlined neural cell outgrowth from the bioink to the microfibrous mesh over multiple z-planes (Fig. S12). In addition, distinct patterns in cytoskeleton arrangement were noticed, as neural cells appeared randomly distributed within the bioink (Fig. 5B and C), while they displayed a more elongated morphology across microfibers. Indeed, neural cells widely adhered and spread along PCL fibers, arranging their cytoskeletons in a preferential aligned direction, as highlighted by the distribution analysis of cell orientations (Fig. 5C). SEM analysis of cellularized constructs confirmed neural cell colonization of MEW scaffolds, where cells highly interacted with microfibers, spreading along fiber surfaces and migrating across scaffold pores (Fig. 5D). In particular, SEM imaging at higher magnification revealed the presence of neural cell clusters embedded in a visible layer of ECM (Fig. 5D).

**Fig. 6:**
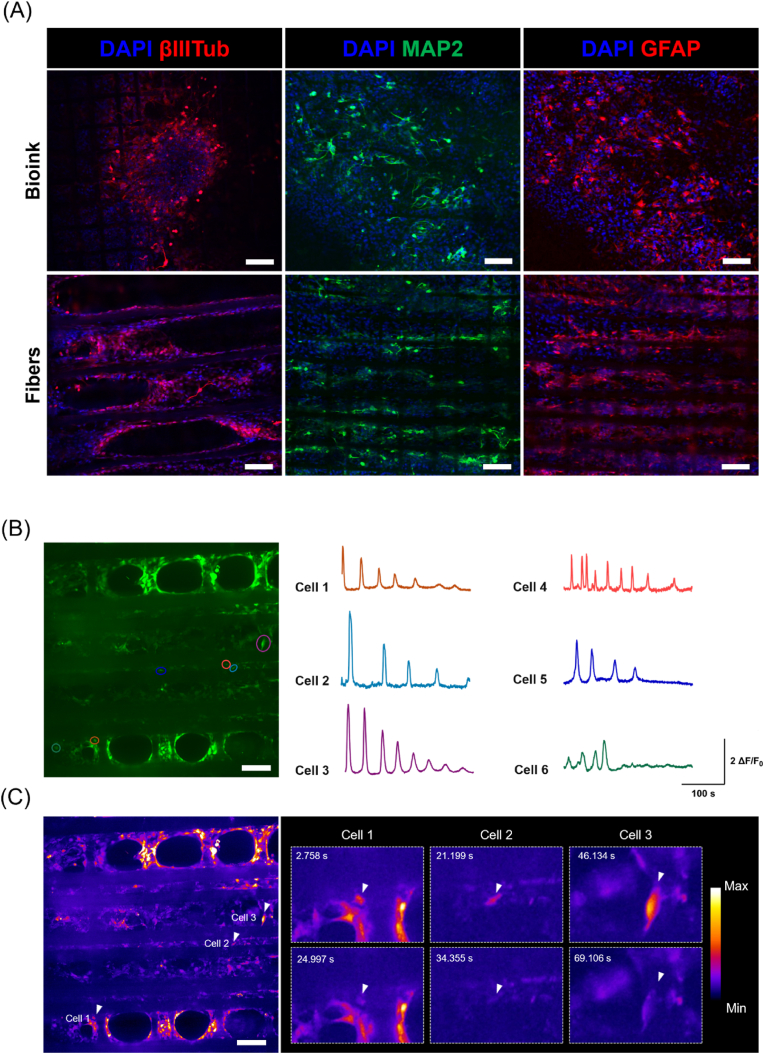
Development of a 3D neural network in 3D bioprinted hybrid constructs. **(A)** Representative immunofluorescence staining for neuronal (βIIITub and MAP2), and astroglial (GFAP) markers in hybrid constructs. Images were acquired in the different compartments of the model (scale bar = 100 μm). **(B)**, **(C)** Functional characterization of 3D bioprinted hybrid constructs by calcium imaging; **(B)** Maximum intensity projection in time from fluorescence time-lapse recordings (left) and cell traces (right) color-coded according to circled ROIs; **(C)** Heatmap of fluorescence from standard deviation Z-projection in time (left) and detail of single time-lapse captures (right) showing changes in fluorescence intensity in selected arrowed cells. Images are false-colored according to fluorescence intensities as described in the calibration bar (scale bar = 100 μm). (For interpretation of the references to color in this figure legend, the reader is referred to the Web version of this article.)

Moreover, immunofluorescence staining for MAP2, βIIITub, and GFAP markers demonstrated NSC differentiation into both neuronal and astroglial cells within the hybrid constructs (Fig. 6A). Differences in neural cell organization and spatial distribution were identified by comparing cells within the bioink and along PCL fibers, where neurons and astrocytes arranged themselves according to fiber orientation. In particular, immunofluorescence staining for the neuronal-specific cytoskeleton proteins revealed extensive neurite outgrowth from neuronal cell bodies with a tendency for directional extension on the fiber surface, suggesting the presence of contact guidance cues mediated by the aligned topography. The differentiation behavior of NSCs was evaluated by Western blot analysis to examine the effect of scaffold topography on NSC fate. Specifically, the expression of phenotype-specific proteins was investigated after 21 days of differentiation culture, compared to the one of cells encapsulated in 3D bioprinted constructs. According to the analysis of relative band intensities (Fig. S13), a higher expression (although not statistically significant) of neuron-specific proteins MAP2 and βIIITub was detected in cells cultured in hybrid constructs compared to 3D bioprinted constructs, while no apparent differences in GFAP expression were observed between the two conditions.

Lastly, the functional maturation of NSC-derived neurons within 3D hybrid constructs was investigated by calcium imaging after three weeks of differentiation culture. Calcium influxes were analyzed by monitoring fluorescence after incubating cells with Flou-4 dye. Live cell imaging allowed for identifying distinct neuron bodies and neurite projections, with numerous interconnections between cell clusters, where spontaneous increases in fluorescence intensity were observed (Fig. 6B and C; Video S1). In particular, time-dependent measurements in selected ROIs showed recurrent calcium transients in cell traces over recording (Fig. 6B), revealing the presence of spontaneous neuronal activity in the neural network.

Supplementary data related to this article can be found online at https://doi.org/10.1016/j.mtbio.2025.102086

The following are the Supplementary data related to this article:

Multimedia component 2 Multimedia component 2

## 4 Discussion

The tridimensional complexity of nervous tissue has unique structural and functional features that are not easy to replicate *in vitro.* Recently*,* advanced biofabrication strategies, relying on additive manufacturing technology, have been employed to create bioengineered neural models characterized by increasing complexity at multiple scale levels [1,61]. Here, we combine extrusion-based 3D bioprinting with the use of aligned microfibrous structures obtained by MEW technology to develop a multi-scaffold system mimicking the composition and anisotropy of the native tissue.

Firstly, a photocrosslinkable GelMA bioink has been formulated by exploiting the thermoresponsive properties of gelatin and the photosensitivity of methacrylate groups, which allow for a physical pre-crosslinking of the pre-gel solution in the extrusion phase followed by a second visible light-induced crosslinking after bioprinting. Indeed, several works have shown the versatility of GelMA polymer for the optimization of photosensitive hydrogels and bioinks, having tunable stiffness and porosity according to polymer concentration [[62], [63], [64], [65], [66]]. In addition, previous studies demonstrated the effectiveness of soft GelMA hydrogel in supporting stem cell viability and growth in 3D bioprinted constructs [67,68]. In particular, it has been observed that GelMA hydrogels having a concentration equal to or less than 5 % successfully promote NSC survival and maturation as they more closely mimic the mechanical characteristics of neural tissues [63,69,70]. Based on these findings, a 5 % GelMA concentration has been selected for the hydrogel formulation.

Specifically, GelMA polymer was synthesized through the direct reaction of gelatin with methacrylic anhydride. The NMR analysis of GelMA proton spectrum revealed the absence of signals attributable to methacrylate groups (i.e., bound to hydroxyl residues), resonating at higher chemical shift than the methacrylamide ones [55], centered between 5.75 ppm and 5.40 ppm. This result suggested that the derivatization mechanism mainly involved the amino groups present on the gelatin chains, while the hydroxyl groups were not derivatized, according to a higher reactivity of the former with respect to the latter in methacryloylation reactions [71]. The comparison of the HSQC maps recorded for raw gelatin and GelMA allowed identifying the amino acids mainly involved in the derivatization process (lysine and hydroxylysine), thus providing useful insights into the derivatization mechanism. Finally, the quantitative analysis carried out in the presence of TMSP for determining DS revealed a very good agreement between results obtained with and without applying solvent presaturation. These data confirmed the possibility of using presaturation for removing the solvent signal and, thus, gaining in signal-to-noise ratio and in the quality of the final spectrum without affecting the quantitative analysis.

Rheological studies were performed to investigate the photopolymerization kinetics and viscoelastic properties of GelMA hydrogel. According to the results (Fig. 1B), GelMA pre-gel displayed stable gel-like behavior and typical shear thinning properties, which are key factors in defining hydrogel printability and shape fidelity as well as ensuring cell survival during the printing process, preventing excessive shear stresses on encapsulated cells [10,72]. In addition, the analysis of photopolymerization kinetics (Fig. 1B) demonstrated the fast sol-gel transition of GelMA solution in response to visible light irradiation, which confers stability to the hydrogel network in physiological conditions due to secondary crosslinking.

Therefore, NSCs were encapsulated into GelMA hydrogel to fabricate neural-like constructs by 3D bioprinting technology. Viability analyses confirmed that NSCs can be successfully bioprinted with no noticeable impact on cell survival (87.86 ± 4.37 % of viable cells after 1 day from the printing process) (Fig. 3A and B) and demonstrated the capability of GelMA bioink in supporting the growth of encapsulated cells over time. Indeed, an increase in cell viability was observed from day 7 to day 14 of culture (Fig. 3C), suggesting NSC proliferation within the printed filaments concurrently with progressive GelMA degradation, according to the timing of the hydrogel stability study (Fig. 1C). Interestingly, NSCs exhibited greater viability when they were embedded in 3D bioprinted constructs compared to 3D hydrogels (Fig. 3D), indicating that the 3D bioprinted configuration could ensure a higher viability of encapsulated cells. This could be probably due to the reduced thickness and higher macroporosity of the printed constructs, which could favor hydrogel perfusion and nutrient exchange [73]. Additionally, NSCs effectively differentiated into mature neurons and astroglial cells, creating extended neural networks within the bioprinted filaments after 21 days of differentiation culture (Fig. 4A). Indeed, immunofluorescence analysis revealed the presence of large MAP2 stain-positive cell aggregates including neuronal cell bodies and pronounced neurite extensions, surrounded by GFAP-expressing cells. The phenotypic characterization by Western blot analyzed the differentiation profile of encapsulated NSCs, confirming early neuronal differentiation after 14 days from RA induction and subsequent NSC differentiation into astroglial cells (Fig. 4B). This is in line with previous studies, which reported the onset of astrogenesis after the main neurogenesis process [[74], [75], [76]]. Hence, these findings demonstrated that 3D bioprinted GelMA hydrogels created an appropriate microenvironment for NSC growth and differentiation into neuronal and glial phenotypes, providing a supporting matrix for neural cell maturation in 3D conditions. Indeed, many studies underlined the potential of natural-based soft bioinks in developing *in vitro* neural microtissues, as they can be accurately designed to reproduce the native ECM environment, due to the high water content, the presence of cell-binding domains, and modifiable mechanical properties [6,77]. These features are essential for the survival of embedded cells and functional for regulating neural cell response and facilitating neuron sprouting and interactions.

However, most hydrogel-based *in vitro* systems resulted in the formation of randomly connected neural networks, thus failing to replicate the inherent organization of neural circuits. Thus, novel strategies have been explored to more accurately mimic the neural microenvironment, providing guidance cues for directional neurite outgrowth and axonal elongation *in vitro* [25,78,79]. In particular, aligned fiber-based substrates have been demonstrated to significantly affect neural cell spatial arrangement and process extensions [29,30,80], but their application in 3D neural culture systems is limited [37,39,78,81]. In addition, most of these studies involve the use of layered constructs obtained by alternating cell-loaded hydrogels on the top or between aligned electrospun nanofibrous mats, resulting in non-uniform cell response over the z-axis.

For this reason, we explored the feasibility of using MEW microfibers to create a biomimetic scaffold with aligned topography and microporosity which could steer neural cell organization in a 3D environment. The combination of MEW with hydrogel-based systems has been proven to support cell growth within multi-material constructs according to specific spatial distribution [82,83]. In our approach, the developed neural bioink has been bioprinted directly onto a melt electrowritten scaffold, to achieve precise cell patterning across PCL microfibers (Fig. 5A). The MEW process has been optimized to improve the printing fidelity and reproducibility, minimizing the fiber diameter and the occurrence of fiber bridging [84]. The obtained PCL scaffolds showed high regularity of the stacked filaments and porosity, resulting in a layered structure with aligned channels, having anisotropic characteristics in terms of morphology and mechanical behavior (Fig. 2C, D, E). The high microporosity of the scaffold design allowed for the optimal integration of the hydrogel within the fibrous structure, resulting in the complete colonization of the hybrid constructs (Fig. 5B andD). Microscopy analysis demonstrated that neural cells were able to extensively migrate within the scaffold pores and fiber interspace, preferentially arranging in alignment with fiber direction (Fig. 5B and C; Fig. 6A; Figure S12). In general, it has been reviewed that cells usually display elongated morphology when cultured on microfibers and aligned fibers, increasing their focal adhesions and reorganizing their cytoskeleton along the main fiber direction [85]. Accordingly, previous works reported neural cell growth in microfibrous scaffolds, having values of fiber diameter and spacing comparable to the ones obtained in this study [51,53,86].

Moreover, immunofluorescence staining confirmed the presence of neuronal and glial cell populations in hybrid constructs after 21 days of differentiation in culture, underlying the effect of fiber alignment on neurite outgrowth (Fig. 6A). In particular, a high number of differentiated cells was observed on the fiber surface and interspace within the microfiber compartment, where pronounced directional extension of cell processes was noticed. On the contrary, neural cells appeared randomly distributed in the bioink compartment, where the effect of fiber topography was minor, probably because of the reduced cell contact area with microfiber and the clustered cell arrangement. Interestingly, the analysis of phenotype-specific markers indicated that the addition of microfibers would also affect NSC differentiation, as a slight increment of neuronal protein expression was found in the case of cells cultured in hybrid constructs compared to the ones encapsulated in 3D bioprinted constructs (Fig. S13). These results suggest that this method could improve the survival and maturation of NSC-derived neurons within the 3D structure. This is consistent with previous reports where enhanced neuronal differentiation was demonstrated in the presence of aligned fibrous components, which more closely reproduce the architecture of neural ECM [22,29,36].

Lastly, intracellular calcium imaging revealed spontaneous neuron signaling after three weeks of differentiation culture, suggesting that NSC-derived neurons were possibly arranging in a functionally active neural network within 3D hybrid constructs (Fig. 6). Time-lapse imaging (Video S1) showed the presence of spontaneous calcium transients across microfibers, where the aligned neural cell arrangement resulted in a directional transmission of neuronal signals, suggesting the importance of scaffold topography in guiding neural cell connectivity [29].

To conclude, we developed an engineered neural tissue replicating some distinctive features of the native microenvironment, including multicellularity and anisotropy, following a novel approach that allowed for the automated fabrication of NSC-laden constructs and their subsequent *in vitro* maturation. The developed system brings several advantages in terms of tissue mimicry and versatility. In particular, the direct bioprinting of NSCs onto the MEW scaffold provides a simple method to obtain fine control of initial cell positioning, ensure high cell viability, and support the establishment of a functional neural network, having aligned 3D arrangement and directional growth. Indeed, the multi-scaffold assembly enabled cells to grow embedded in a matrix that closely resembles the extracellular environment while experiencing the anisotropic cues given by the MEW scaffold. Hence, this platform holds the potential to develop more sophisticated tissue models for instance by integrating multiple neuronal subtypes, which can be bioprinted according to defined geometries recreating functional neural circuits. In this regard, the use of neural progenitor cells derived from human induced pluripotent stem cells would be a promising alternative for the generation of specific neuronal populations, while also representing a significant step forward for model validation, both in terms of biological relevance and applicability. Currently, self-assembled 3D models, such as organoids or assembloids, have been typically exploited to study inter-cellular interactions occurring in neural development and diseases [1,[87], [88], [89]], but their use involves many challenges, including poor control over structural characteristics, size-dependent nutrient diffusion, and low reproducibility [90]. In contrast, our engineering approach offers the possibility of creating compartmentalized constructs with tunable shape and size, which would be useful for studying cellular behavior under physiological and pathological conditions. Due to its modularity, the described methodology supported the establishment of co-culture systems, allowing for investigating intercellular interactions and neuronal connectivity within an *in vivo*-like microarchitecture. In addition, this approach would be likely applied to replicate CNS injuries *in vitro*. For instance, CNS trauma could be possibly modeled via the induction of transection and/or stretch injuries in the central microfibrous compartment. As such, the described multi-scaffold system would be exploited to investigate the pathological events occurring after neural tissue injury and test regenerative treatments.

## 5 Conclusions

Overall, this study provides an alternative approach for the development of functional *in vitro* models of neural tissue, combining the use of soft hydrogels and aligned microfibrous structures to support NSC growth and differentiation towards mature neural cell populations. The use of additive manufacturing technology allowed the design of specific microscale architecture, which was shown to highly influence neural cell arrangement and activity, thereby exploiting the advantages of aligned fiber-based substrates in a biomimetic 3D configuration. Lastly, this multi-scaffold system is characterized by structural stability and easy handling, thus providing a versatile platform for long-term studies on CNS functions and pathology.

## CRediT authorship contribution statement

**Cecilia Traldi:** Writing – original draft, Visualization, Methodology, Investigation, Data curation, Conceptualization. **Vanessa Chiappini:** Methodology, Investigation. **Silvia Chasseur:** Methodology, Investigation. **Federica Aiello:** Writing – review & editing, Methodology, Investigation. **Marina Boido:** Writing – review & editing, Supervision, Project administration, Methodology, Investigation, Funding acquisition, Conceptualization. **Chiara Tonda-Turo:** Writing – review & editing, Supervision, Project administration, Methodology, Investigation, Funding acquisition, Conceptualization.

## Declaration of competing interest

The authors declare the following financial interests/personal relationships which may be considered as potential competing interests: Chiara Tonda-Turo reports financial support was provided by 10.13039/501100024370Ministry of Education and Merit. If there are other authors, they declare that they have no known competing financial interests or personal relationships that could have appeared to influence the work reported in this paper.

## Data Availability

Data will be made available on request.
