# Supplementary material for: 3D in vitro modeling of neural microenvironment through a multi-scaffold assembly approach
Source: Mater Today Bio. 2025 Jul 14;33:102086. doi: 10.1016/j.mtbio.2025.102086 (PMC12303067; doi:10.1016/j.mtbio.2025.102086)
Supplement: Multimedia component 2 [file mmc2.docx]

**Supplementary material**

**
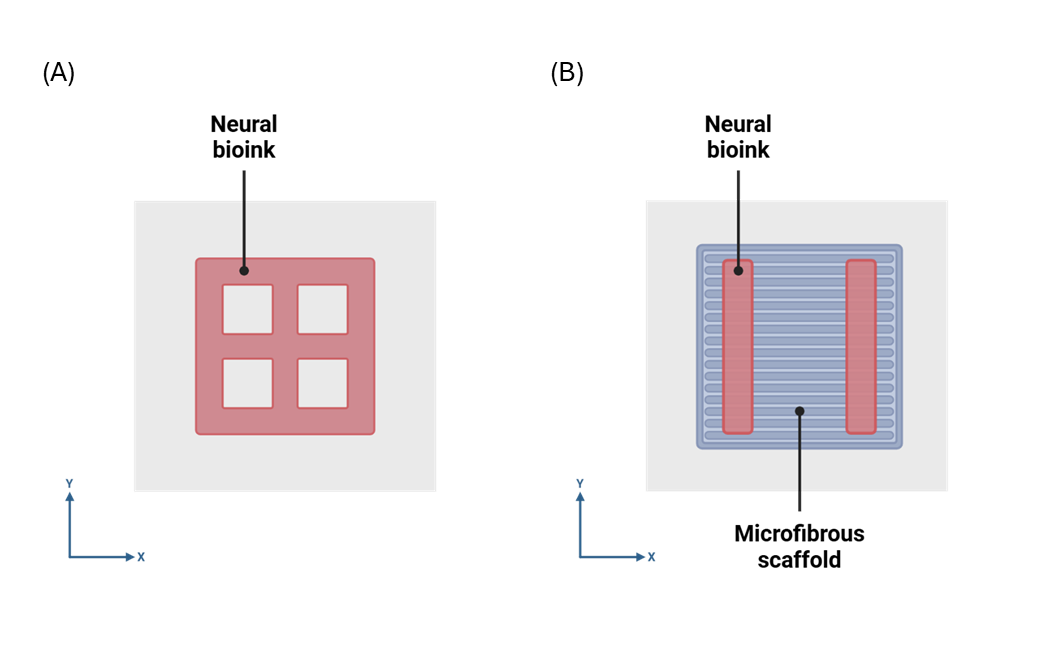
**

**Figure S1.** 3D bioprinting of NSC-laden constructs. Schematic description of the two printing geometries designed to bioprint NSC-laden constructs according to a grid pattern to perform primary studies on NSC viability and differentiation **(A)** and 3D bioprinted hybrid constructs obtained by directly bioprinting neural bioink onto MEW microfibrous scaffolds **(B)** (Created with BioRender.com).

**
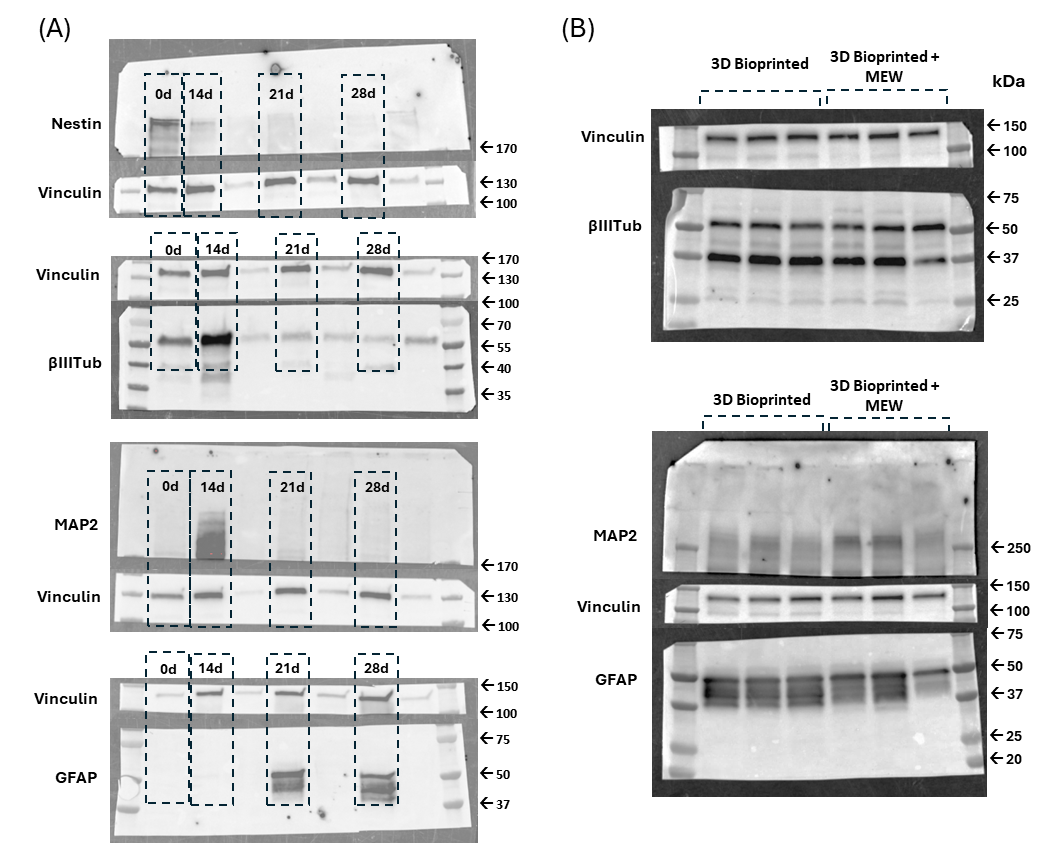
**

**Figure S2.** Uncropped western blot membranes. (A) Western blot analysis for the expression of nestin, βIIITub, MAP2, and GFAP in 3D bioprinting constructs at 0, 14, 21, and 28 days of differentiation culture. Dotted boxes are referred each day shown in Fig. 4. (B) Western blot used to compare βIIITub, MAP2, and GFAP expression in 3D bioprinted constructs (3D Bioprinted) vs 3D bioprinted hybrid constructs (3D Bioprinted + MEW) at 21 days of differentiation culture, referred to Fig. S11.


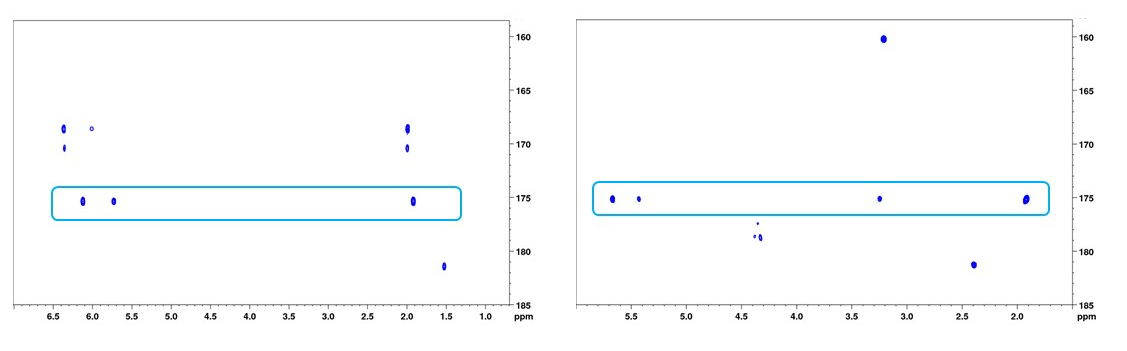


**Figure S3.** Expansion of HMBC (700 MHz, D_2_O, 298 K) maps of (left) methacrylic anhydride and (right) GelMA, with long-range ^1^H-^13^C correlations confirming the derivatization process


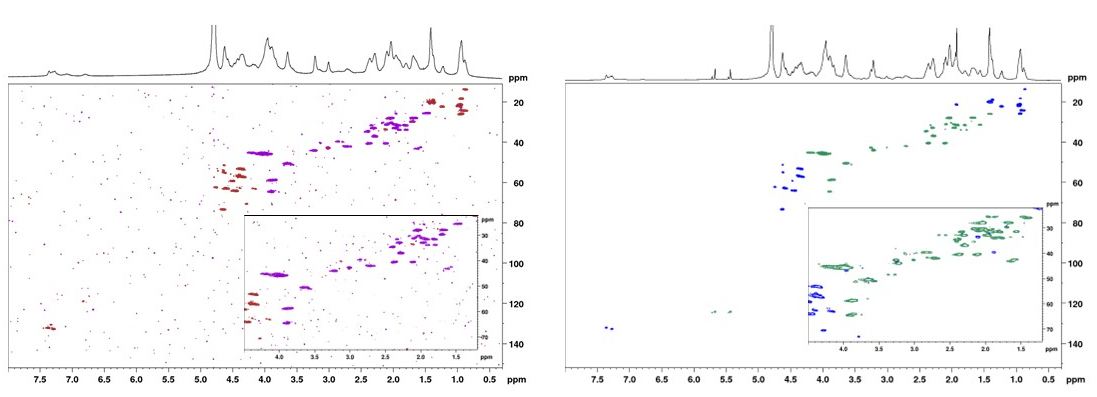


**Figure S4.** HSQC (700 MHz, D_2_O, 298 K) maps of (left) underivatized gelatin and (right) GelMA, with expansion showing the new signals found in GelMA as a consequence of derivatization.


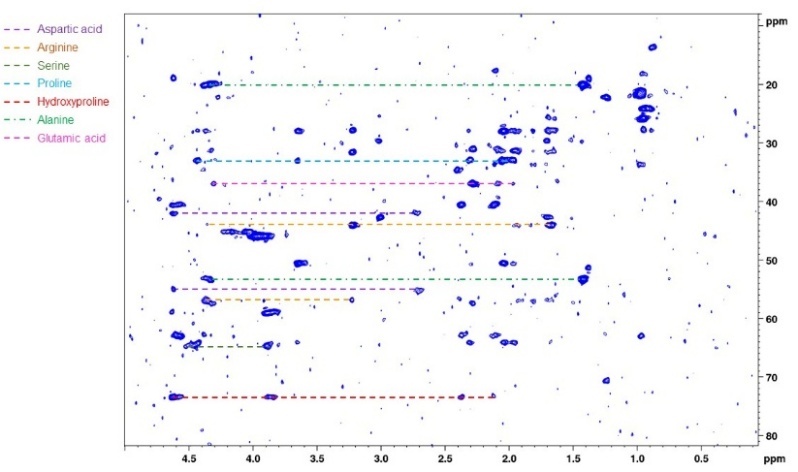


**Figure S5.** HSQC-TOCSY (700 MHz, D_2_O, 298 K) map of underivatized gelatin with some of the amino acids identified.

**
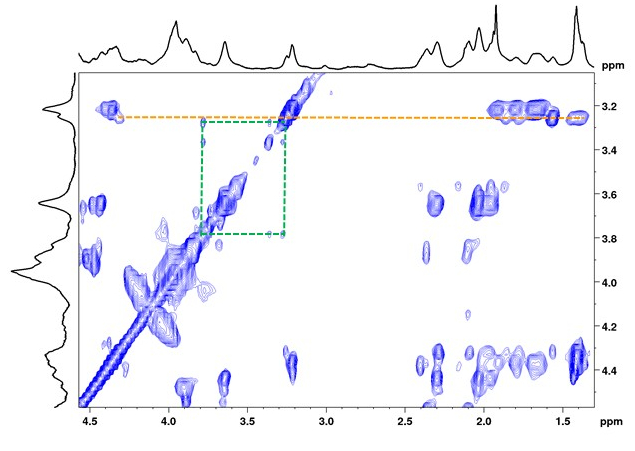
**

**Figure S6.** Expansion of TOCSY (700 MHz, D_2_O, 298 K, mixing time = 80 ms) map of GelMA; the dotted lines indicate the correlations between the protons of modified lysine (yellow) and hydroxylysine (green), respectively.

**
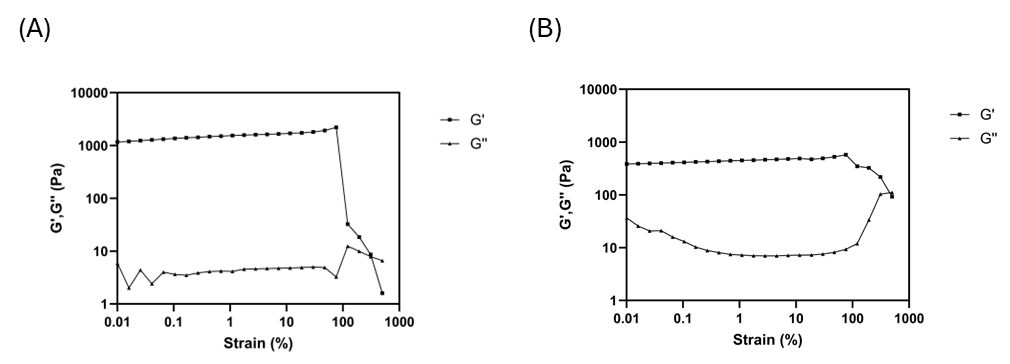
**

**Figure S7.** Rheological characterization of GelMA hydrogel. Strain sweep tests of GelMA hydrogel after visible light-induced photocrosslinking **(A)** and after temperature-induced physical gelation at 20°C **(B)**.


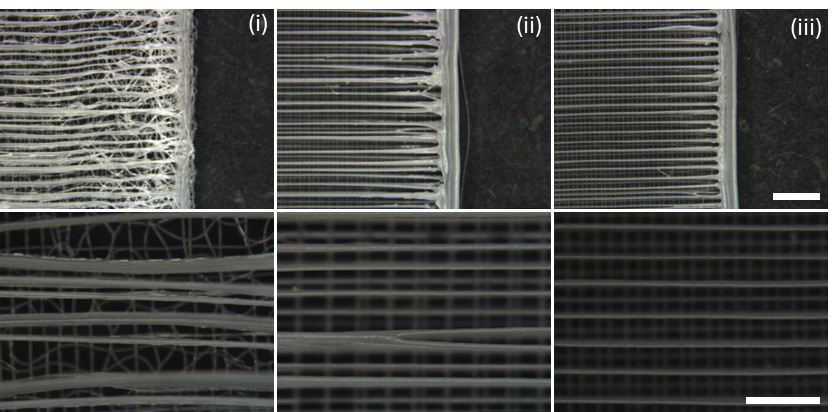


**Figure S8**. Morphological characterization of aligned microfibrous scaffolds. Representative optical microscopy images for samples fabricated with different sets of parameters: i (4500 V voltage, 3% flow rate), ii (3030 V voltage, 1% flow rate), iii (3030 V voltage, 0.8% flow rate); (top row scale bar = 1 mm, bottom row scale bar = 500 μm).


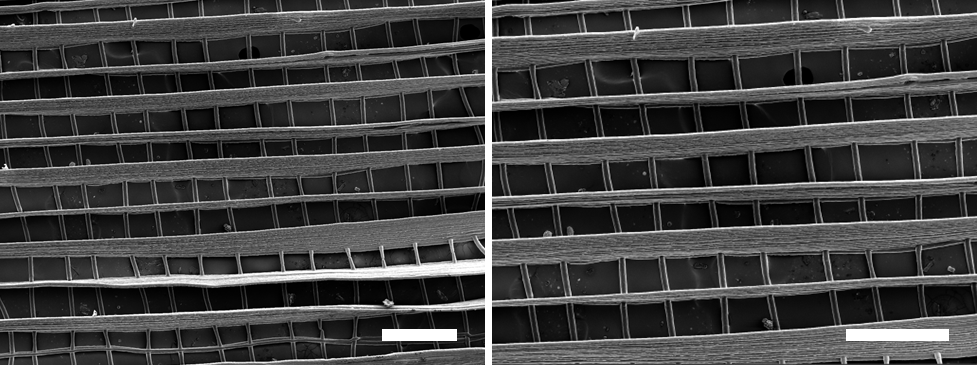


**Figure S9**. Morphological characterization of aligned microfibrous scaffolds. Representative SEM microscopy images of the MEW scaffolds fabricated with the optimal set of parameters (scale bar = 200 μm).


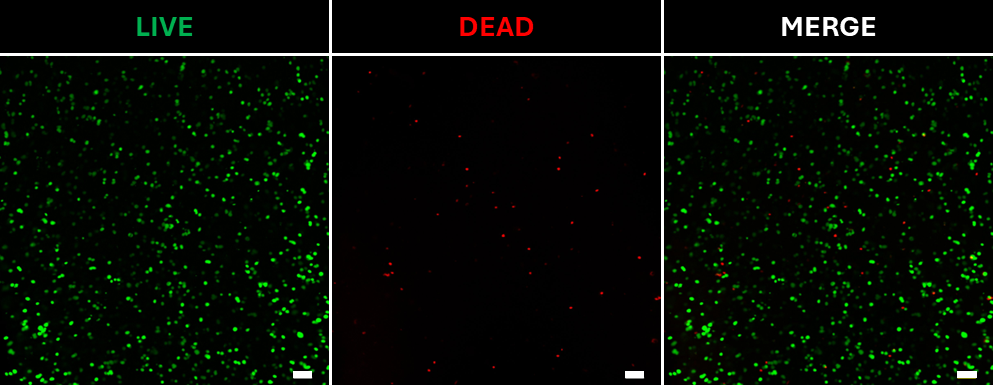


**Figure S10.** Investigation of NSC viability in 3D bioprinted constructs. Representative fluorescence images of LIVE/DEAD® assay in 3D bioprinted constructs after 1 day from the printing process (10X magnification; scale bar = 100 μm).


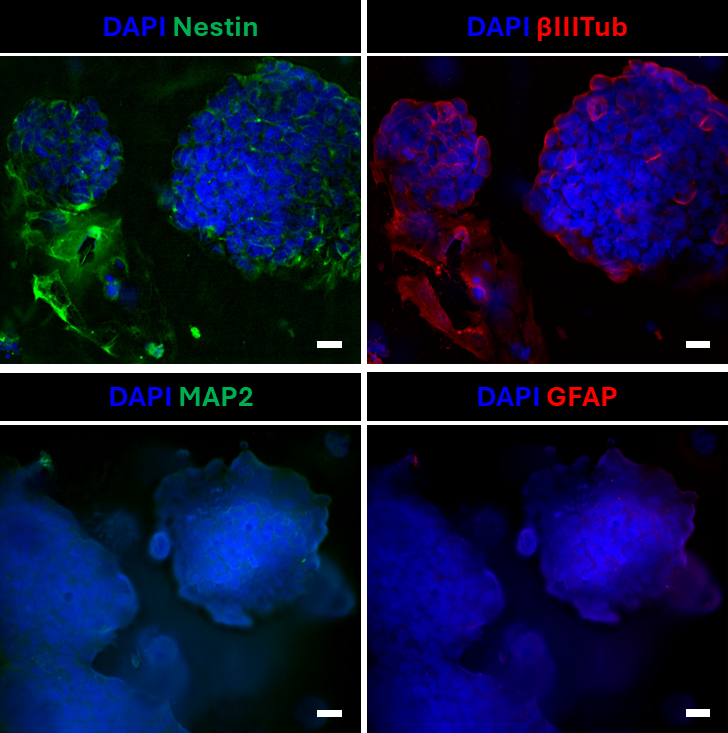


**Figure S11.** Investigation of NSC differentiation in 3D bioprinted constructs. Representative immunofluorescence images for stemness-related (nestin), neuronal (βIIITub and MAP2), and astroglial (GFAP) markers in NSC-laden 3D bioprinted constructs at day 0 of differentiation culture (scale bar = 20 μm).


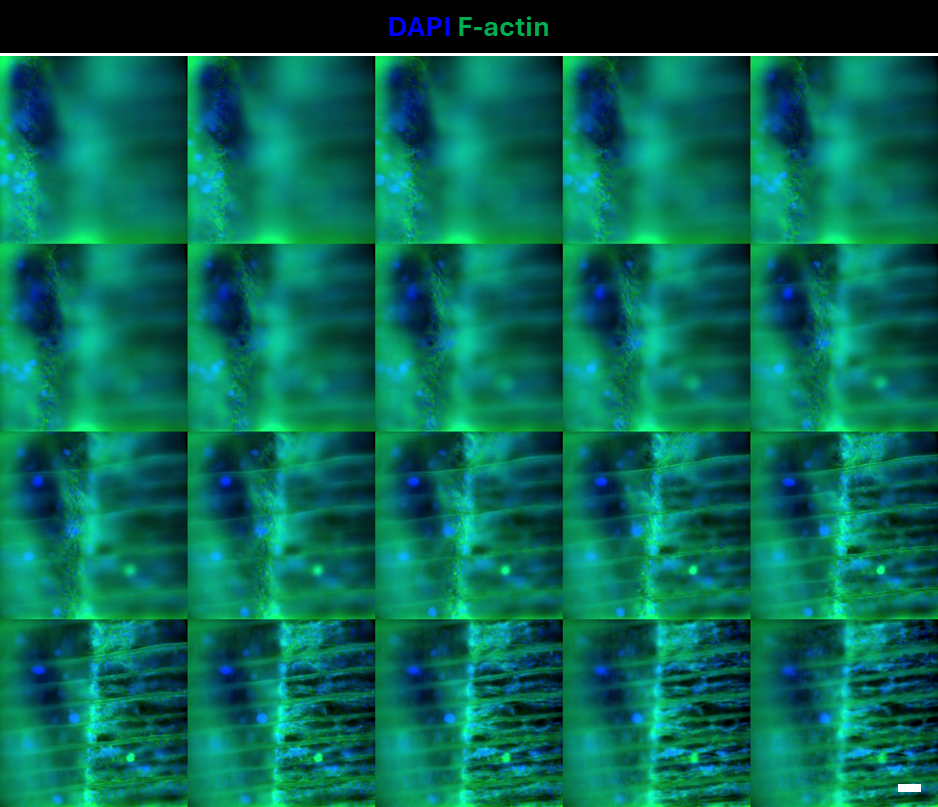


**Figure S12.** Fluorescence staining for cell cytoskeletons in 3D bioprinted hybrid constructs. Z-stack image sequence acquired at the bioink/fiber interface after 21 days from differentiation induction. The images were captured every 10 μm in 200 μm thickness from the higher layers (hydrogel z-planes) to the bottom layers (fiber z-planes) of the hybrid constructs (scale bar = 100 μm).


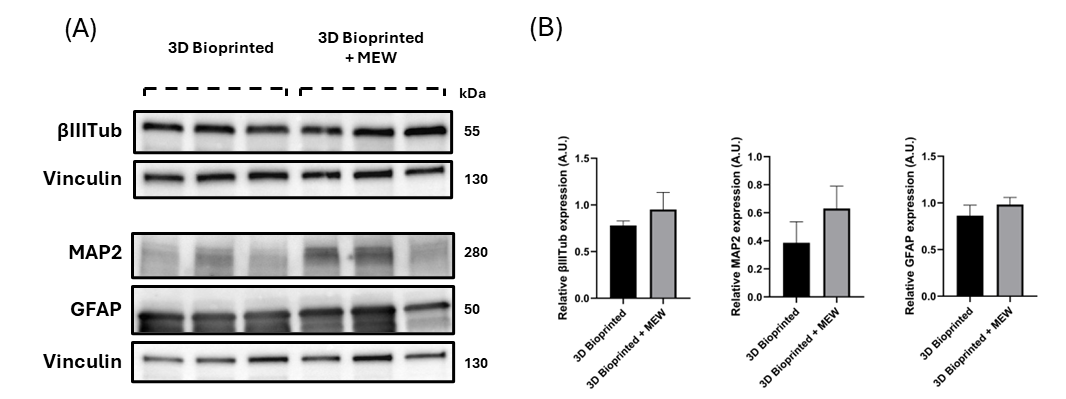


**Figure S13.** Comparison of NSC differentiation in 3D bioprinted constructs (3D Bioprinted) and 3D bioprinted hybrid constructs (3D Bioprinted + MEW). **(A)** Representative densitometries and **(B)** protein level quantifications of βIIITub, MAP2, and GFAP at 21 days of differentiation culture. Data are presented as mean ± SD (n = 3 biological replicates).

**Table S1.** ^1^H (700 MHz, D_2_O, 298 K) and ^13^C (175 MHz, D_2_O, 298 K) characterization data of the main amino acids found in underivatized gelatin.

| amino acid | fragment | δ_H_ (ppm) | δ_C_ (ppm) | amino acid | fragment | δ_H_ (ppm) | δ_C_ (ppm) |
| --- | --- | --- | --- | --- | --- | --- | --- |
| alanine | α-CH | 4.34 | 53.3 | leucine | α-CH | 4.37 | 56.9 |
|  | β-CH_3_ | 1.41 | 20.0 |  | β-CH_2_ | 1.63 | 42.9 |
| arginine | α-CH | 4.36 | 56.9 |  | γ-CH | 1.68 | 27.9 |
|  | β-CH_2_ | 1.94/1.80 | 31.5 |  | δ-CH_3_ | 0.89 | 24.2 |
|  | γ-CH_2_ | 1.66 | 27.7 |  | δ-CH_3_ | 0.94 | 25.6 |
|  | δ-CH_2_ | 3.23 | 43.9 | lysine | α-CH | 4.37 | 56.8 |
| asparagine | α-CH | 4.62 | 54.6 |  | β-CH_2_ | 1.90/1.80 | 33.6 |
|  | β-CH_2_ | 2.86/2.79 | 39.6 |  | γ-CH_2_ | 1.43 | 25.3 |
| aspartic acid | α-CH | 4.64 | 54.8 |  | δ-CH_2_ | 1.68 | 29.6 |
|  | β-CH_2_ | 2.70 | 42.0 |  | ε-CH_2_ | 3.01 | 42.6 |
| glycine | α-CH_2_ | 3.97 | 45.9 | phenylalanine | α-CH | 4.74 | 62.3 |
| glutamic acid | α-CH | 4.39 | 56.7 |  | δ-CH (Ar) | 7.29 | 132.8 |
|  | β-CH_2_ | 2.09/1.97 |  |  | ε-CH (Ar) | 7.37 | 132.2 |
|  | γ-CH_2_ | 2.27 | 36.9 |  | ζ-CH (Ar) | 7.33 | 130.6 |
| glutamine | α-CH | 4.39 | 56.7 | proline | α-CH | 4.43 | 64.1 |
|  | β-CH_2_ | 2.16/2.03 | 30.1 |  | β-CH_2_ | 2.30/1.96 | 32.9 |
|  | γ-CH_2_ | 2.42 | 34.6 |  | γ-CH_2_ | 2.03 | 27.9 |
| hydroxylysine | δ-CH | 3.89 | 70.5 |  | δ-CH_2_ | 3.65 | 50.6 |
| hydroxyproline | α-CH | 4.58 | 62.7 | serine | α-CH | 4.49 | 59.1 |
|  | β-CH_2_ | 2.37/2.11 | 40.5 |  | β-CH_2_ | 3.89 | 64.6 |
|  | γ-CH | 4.63 | 73.1 | threonine | α-CH | 4.39 | 62.6 |
|  | δ-CH_2_ | 3.84 | 58.7 |  | β-CH | 4.27 | 70.3 |
| isoleucine | α-CH | 4.14 | 63.1 |  | γ-CH_3_ | 1.23 | 22.2 |
|  | β-CH | 1.90 | 39.3 | tyrosine | δ-CH (Ar) | 7.10 | 131.6 |
|  | γ-CH_2_ | 1.45 | 25.4 |  | ε-CH (Ar) | 6.82 | 119.0 |
|  | γ-CH_3_ | 0.92 | 18.3 | valine | α-CH | 4.18 | 62.8 |
|  | δ-CH_3_ | 0.87 | 13.6 |  | β-CH | 2.11 | 33.4 |
|  |  |  |  |  | γ-CH_3_ | 0.95 | 21.5 |

**Table S2.** ^1^H (700 MHz, D_2_O, 298 K) and ^13^C (175 MHz, D_2_O, 298 K) characterization data of modified amino acids detected in GelMA.

| Modified amino acid | fragment | δ_H_ (ppm) | δ_C_ (ppm) |
| --- | --- | --- | --- |
| lysine | α-CH | 4.33 | 57.2 |
|  | β-CH_2_ | 1.86/1.77 | 33.9 |
|  | γ-CH_2_ | 1.58 | 31.4 |
|  | δ-CH_2_ | 1.40 | 25.8 |
|  | ε-CH_2_ | 3.25 | 42.7 |
| hydroxylysine | δ-CH | 3.78 | 73.0 |
|  | ε-CH_2_ | 3.37-3.28 | 48.4 |
